# Maternal Separation Followed by Chronic Mild Stress in Adulthood Is Associated with Concerted Epigenetic Regulation of AP-1 Complex Genes

**DOI:** 10.3390/jpm11030209

**Published:** 2021-03-16

**Authors:** Lene Lundgaard Donovan, Kim Henningsen, Anne Flou Kristensen, Ove Wiborg, John Dirk Nieland, Jacek Lichota

**Affiliations:** 1 Neurobiology Research and Drug Delivery Group, Department of Health Science and Technology, Aalborg University, 9220 Aalborg Øst, Denmark; lene.donovan@nru.dk (L.L.D.); anneflou86@gmail.com (A.F.K.); ow@hst.aau.dk (O.W.); 2 Department of Biomedicine-Dandrite, Takeuchi Team, Aarhus University, 8000 Aarhus C, Denmark; kh@biomed.au.dk; 3 Molecular Pharmacology Group, Department of Health Science and Technology, Aalborg University, 9220 Aalborg Øst, Denmark; jdn@hst.aau.dk

**Keywords:** depression, epigenetics, maternal separation, chronic mild stress

## Abstract

Depression is one of the most prevalent mental diseases worldwide. Patients with psychiatric diseases often have a history of childhood neglect, indicating that early-life experiences predispose to psychiatric diseases in adulthood. Two strong models were used in the present study: the maternal separation/early deprivation model (MS) and the chronic mild stress model (CMS). In both models, we found changes in the expression of a number of genes such as *Creb* and *Npy*. Strikingly, there was a clear regulation of expression of four genes involved in the AP-1 complex: *c-Fos*, *c-Jun*, *FosB*, and *Jun-B*. Interestingly, different expression levels were observed depending on the model, whereas the combination of the models resulted in a normal level of gene expression. The effects of MS and CMS on gene expression were associated with distinct histone methylation/acetylation patterns of all four genes. The epigenetic changes, like gene expression, were also dependent on the specific stressor or their combination. The obtained results suggest that single life events leave a mark on gene expression and the epigenetic signature of gene promoters, but a combination of different stressors at different life stages can further change gene expression through epigenetic factors, possibly causing the long-lasting adverse effects of stress.

## 1. Introduction

Depression is one of the most costly and prevalent mental diseases worldwide. It is predicted to be the leading cause of disease burden worldwide in 2030 [1]. Even though this disease can be cured when detected early, many people do not seek help due to stigmatization or limited access to specialized health service. Only a very small portion of people with depression are diagnosed and undergo treatment. The existing treatment is unfortunately not very effective. Only about 20% of patients receiving cognitive behavioral therapy and 30% of those receiving antidepressants alone will be cured by the treatment [2]. 

Early-life adverse experiences have been implicated as predisposing factors for psychiatric diseases in adulthood [3], since patients often have a history of childhood neglect [4,5]. Even though the risk of disease increases immediately after exposure to these stressful events, the effects can persist over the life course. Rodent and primate studies have shown that the very early postnatal period is of specific importance during development. The mother–pup relationship at this stage is key to normal development, while disturbances can have profound long-lasting effects on behavior in adulthood [6]. A disturbed Hypothalamic–Pituitary–Adrenal (HPA) axis was suggested as a potential underlying cause of the resulting pathophysiology. Several studies have shown that early-life stress induces a long-lasting hyperactive HPA axis [7]. Henningsen et al., 2012 described the effect of maternal care on the response to chronic mild stress (CMS) in the adult rat. A low level of maternal care, as measured by five different types of behavior, predisposed rats to higher stress susceptibility. Moreover, it was documented that CMS increased the corticosterone response [8]. 

It is well established that genetic factors are implicated in the etiology of depression. Depression, in terms of major depressive disorder (MDD), runs in families. People in these families have therefore significantly increased risk of having a relative with the disease [9]. In the present study, we chose to use the maternal separation/early deprivation model of early-life stress to test our hypothesis that early-life stressors such as disturbances of this familiar link may contribute to depression in adulthood. A large number of studies have used this model to study the long-lasting effects of this type of early-life deprivation on adult behavior (reviewed in [10]). These studies have shown that rats exposed to maternal separation as pups permanently maintained high anxiety-like behavior [11] that elicited a depression-like syndrome [12].

There are several studies suggesting a correlation between external environmental factors and cellular responses through epigenetics factors. DNA methylation was investigated in rats and humans as a main epigenetic factor playing a role in translating childhood adversity into a depressive phenotype. It was shown that DNA methylation changes occurred both in the hippocampus on the gene promoter of glucocorticoid receptor (GR, NR3C1), spreading throughout the 7 million base-long genomic locus on chromosome 18 [13], and systemically, as shown by T cell analysis [6]. These epigenetic changes also included histone modifications [13] and were responsible for a changed expression of GR (and other) gene, thereby suggesting that these aberrations were causing depression in adult life. The epigenetic events related to early-life adversity first shown in rats [14] are translatable to humans, as suggested by several studies. Changes in DNA methylation were shown to be associated with child abuse [15] and early-life socio-economic position in humans [16]. It is therefore highly relevant to use animal models to study mechanisms related to depression.

The present study was conducted on rats to allow comparisons with several important articles in the field. Our two main aims were: (1) to investigate whether exposure of rats to maternal separation (MS) would predispose the offspring to increased stress susceptibility as measured by sucrose intake after exposure to CMS; (2) to determine whether a potential effect of maternal separation was associated with distinct epigenetic pattern(s). 

## 2. Materials and Methods

### 2.1. Animals and Treatment 

The experiments were conducted on 35 timed-pregnant Wistar rats (Taconic MB), arrived at the facility when they were 14 days in the gestation period. Dams were individually housed and maintained under standard laboratory conditions (relative humidity 40% ± 5%, light/dark cycle 6.00–18.00, food and water ad libitum). Pups were assigned to one of two rearing conditions: (1) standard facility conditions with handling of the pups twice a week during routine cage changes; (2) daily 180 min MS from p2 to p14, including both starting and final day. During separation, each dam was removed from the home cage and placed in an adjacent identical cage until the end of the separation period. Pups were thereafter removed as complete foster litters and placed into an empty cage that was transferred to an adjacent temperature-regulated room to make sure that the pups did not suffer from hypothermia. At three weeks of age, males were selected and placed in groups of 3–4 rats in a standard cage (females were not used in the following experiments, due to potential issues with the estrous cycle affecting the readouts, thus increasing variations and ultimately demanding a higher number (n) of experimental subjects). 
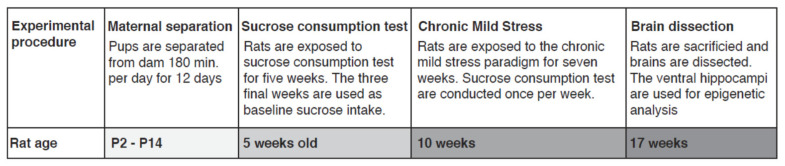


A total of 85 males were used in the CMS experiment. The rationale behind the CMS model is to apply a number of successive microstressors, in an unpredictable order so to prevent habituation, to induce an anhedonic-like state in rats after chronic stress exposure [17]. On the basis of sucrose intake in the three final baseline tests (see below), animals were divided into two groups and placed in separate rooms. One group was exposed to 7 weeks of chronic mild stressors (MS/CMS), and the other was left unchallenged (MS + control). The unchallenged group was food- and water-deprived 14 h before the sucrose consumption test. The stress protocol consisted of seven different stress conditions, each lasting 10 to 14 h: one period of intermittent illumination, stroboscopic light, grouping, food or water deprivation, two periods of soiled cage and no stress, and three periods of 45° cage-tilting [8].

A summary of the different paradigms and animal groups is presented in the Table 1.

### 2.2. Sucrose Consumption Test

One of the major symptoms in clinical depression is ’anhedonia’, which is characterized by a decreased ability to feel pleasure in activities that are normally pleasurable. In rodents, sucrose consumption is used as a behavioral readout to quantify the hedonic state and is interpreted as a measure of reward sensitivity corresponding to the ability of humans to feel pleasure [17]. At five weeks of age, rats were placed in individual cages and trained to consume a palatable sucrose solution (1.5%). The training continued for 5 weeks; the sucrose test was conducted twice a week during the first 3 weeks and once a week during the last 2 weeks. The animals were food- and water-deprived 14 h before the test, which consisted of 1 h of free access to a bottle containing a sucrose solution. Sucrose consumption was measured by weighing the bottles at the beginning and at the end of the test. During the stress period, the sucrose consumption test was performed once a week. Independent of the sucrose consumption test, water intake was monitored once a week by weighing the water bottles before and after a 24 h period. Rats with a mean sucrose intake below 8.5 g were designated as innate low drinkers and were not used for further experiments. 

Baseline sucrose consumption was defined as the mean sucrose consumption during three sucrose tests conducted before stress initiation. Rats were allocated into experimental groups based on their baseline sucrose intake. The maternally separated and control rats were divided into control and stress groups. Following exposure to stress, rats were characterized as anhedonic (characterized by over 30% within-subject decrease in sucrose intake) or resilient (characterized by less than 10% within-subject decrease in sucrose intake). Rats not corresponding to either criterion were excluded from further experiments. Student’s t-test was used to compare the mean sucrose intake of rats exposed to MS compared to controls before MS rats were divided in the control and CMS groups. The sucrose data for the groups assigned to the epigenetic studies were analyzed using a one-way ANOVA approach. This was followed by planned comparisons of the predicted means to compare the levels of the selected effect.

When combining MS and CMS, the following experimental groups were investigated (*n* = 6): (1) Control; (2) MS; (3) CMS; (4) Anhedonic MS/CMS; (5) Resilient MS/CMS (Table 1).

### 2.3. Tissue Preparation

Animals were sacrificed at the age of 16–17 weeks. Following decapitation, the brains were swiftly removed, and the ventral hippocampi dissected and snap-frozen in liquid nitrogen. All samples were stored at −80 °C until further use. The hippocampi from the right hemisphere were used for expression analysis, whereas the hippocampi from the left hemisphere of the same animals were used for epigenetic analysis.

### 2.4. Expression Analysis

Total DNA and RNA was isolated from the right hippocampi by the AllPrep DNA/RNA Mini Kit (Qiagen) according to the manufacturer’s protocol. 

For the experiment, 60 ng of DNase-treated RNA was reverse-transcribed using the RevertAidTM Premium First Strand cDNA Synthesis Kit (Fermentas) according to the manufacturer’s protocol. We used 1 µL of cDNA for qPCR, with Brilliant II SYBR Green QPCR Master Mix and 0.5 μM of each primer. The reactions were run in an Mx3000PTM QPCR System (Stratagene) for 40 cycles: 95 °C for 30 s, 60 °C for 30 s. All samples were run in duplicates, and the *Actb* gene was used for normalization. The Pfaffl method was used for quantification [18]. Statistical analysis was done by One-way ANOVA, followed by Tukey’s post hoc test. Statistical analyses were performed using the GraphPad Prism 5.0 software.

Primers used: Actb 5′AAGGGACACCGTAGAGGGGTGGAGC 3′, 5′ CAGGAGCGTGCCCACGAGTGTCTAC 3′; Creb 5′CAGTTCAAGCCCAGCCACAGATTGC 3′, 5′CATGGACCTGGACTGTCTGCCCATT 3′; Npy 5′CATGGCCAGATACTACTCCGCTCTGCGA3′, 5′AGCCTTGTTCTGGGGGCATTTTCTGTGC 3′; c-Fos 5′GGTCACAGAGCTGGAGCCCCTGTGC 3′, 5′TCGTTGCTGCTGCTGCCCTTTCGGT 3′; c-Jun 5′CCTCAACGCCTCGTTCCTCCAGTC 3′, 5′CGTGAGAAGGTCCGAGTTCTTGGCT 3′; FosB 5′GTCTTCGGTGGACTCCTTCGGCAGT 3′, 5′GTCCTGGCTGGTTGTGATTGCGGTG 3′; JunB 5′GCTGTCAAGTACTGCCGGCCTCCTA 3′, 5′GTGTCCGTATGGAGCAAGGGAGGCT 3′.

### 2.5. Chromatin Immunoprecipitation

Native chromatin immunoprecipitation (NChIP) was performed as previously described [19]. In brief, a single hippocampus was disrupted by knife homogenization, and the cells were lysed. Micrococcocal nuclease (MNase) (Sigma Aldrich) was used to digest chromatin followed by nucleosome extraction by overnight dialysis. Antibodies raised against the desired histone modification (H3K4me3 (ab8580, Abcam), H3K9me3 (ab8898, Abcam), H4ac antibody (06-866, Upstate/Millipore) were added to the nucleosomes for overnight incubation. Protein A-coated magnetic beads (Life Technologies) were added for precipitation of the antibody–nucleosome complexes. Rabbit serum was used as control (mock). The beads were washed, the complexes were eluted, and DNA was purified by the QIAquick PCR purification kit (Qiagen) according to the manufacturer’s protocol. Then, 1 µL of eluted DNA and the Luminaris qPCR Master Mix (Thermo Scientific) with 0.5 µM of each primer were used in a qPCR reaction. The reactions were run in a Mx3000PTM QPCR System (Stratagene) for 40 cycles: 95 °C for 30 s, 60 °C for 30 s, and 72 °C for 30 s. All samples were run in triplicates. *Actb* and *Hist1H2BA* genes were used for normalization purpose.

The Pfaffl method was used for quantification [18]. Statistical analysis was performed on log-transformed data by one-way ANOVA, followed by Tukey’s post hoc test. Statistical analyses were performed using the GraphPad Prism 5.0 software.

Primers used: *Actb* 5′GTGGCACCACCATGTACCCAGGCAT3′, 5′ACTACAGGGCTGACCACACCCCACT3′; *c-Fos* 5′TTACTACGTCATGAGCGGAACAGAG3′, 5′AGGTGAAAGTTACAGACTGAGACGG3′; *Fos-B* 5′GTCCAAAGAAATGAGGGAGAGCAGA3′, 5′TTGTGGGTTTCCTTTTTGCAAGACT3′; *c-Jun* 5′AAAATAGCCCATGATGTCACCCCAA3′, 5′CATTACCTCATCCCGTGAGCCTTC3′; *Jun-B* 5′TGGCTATGAGATCTCTGTACACTGC3′, 5′CTTCCCTGTTTTCTGGGGTCTTCTA3′; *Hist1H2BA* 5′TCCAAACACTTTCCATATCCCCACT3′, 5′TGGTGATAAGCCTGGAACACAGATT3′.

## 3. Results and Discussion

**Figure 1:**
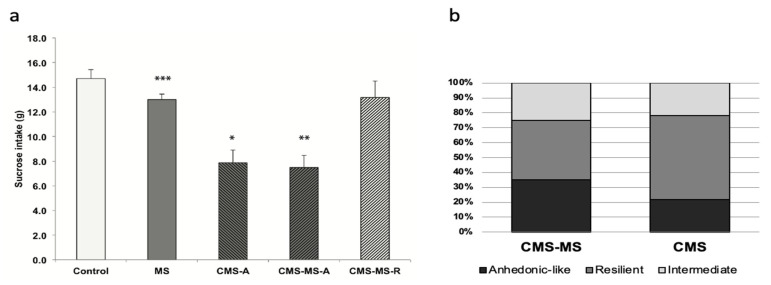
(**a**) Sucrose intake of rats in all the experimental groups. Control: unchallenged (*n* = 10), MS: *n* = 10, CMS-A: animals exposed to chronic mild stress, exhibiting anhedonia (*n* = 6), CMS-MS-A: *n* = 8, CMS-MS-R: *n* = 8. Data are shown as mean group sucrose intake +/− SEM, comparing each group with the control group * = *p* < 0.05, ** = *p* < 0.02, *** = *p* < 0.001; (**b**) Increased stress susceptibility of rats shown as percentage of animals defined as anhedonic-like, resilient, or intermediate in the CMS or CMS-MS group after seven weeks of CMS.

The animals were subjected to the MS protocol in early life, followed by CMS in adulthood, and sucrose intake was measured once a week from age 5 weeks, as a proxy for depression-like behavior. A comparison of baseline sucrose intakes (before MS rats were divided in control and CMS groups) showed a significant effect of MS alone on baseline sucrose intake (*p* = 0.009), with rats exposed to MS showing significantly decreased sucrose intake. The biggest anhedonic-like effect measured by the sucrose intake test was observed after the induction of CMS, regardless of MS, as shown by comparison with the control group (CMS (*p* = 0.04) and MS/CMS-A (*p* = 0.02)). As expected, the resilient group (MS/CMS-R) exhibited a sucrose intake similar to that of the control group and significantly different from that of the anhedonic-like MS/CMS-A group (*p* = 0.01) (Figure 1a). Furthermore, we evaluated the effect of CMS by determining the number of rats with different phenotypes in groups exposed to MS/CMS or CMS alone. The fraction of anhedonic-like rats was larger in the MS/CMS group compared to the CMS group, whereas the fraction of resilient rats was larger in the CMS group compared to the MS/CMS group (Figure 1b).

**Figure 2:**
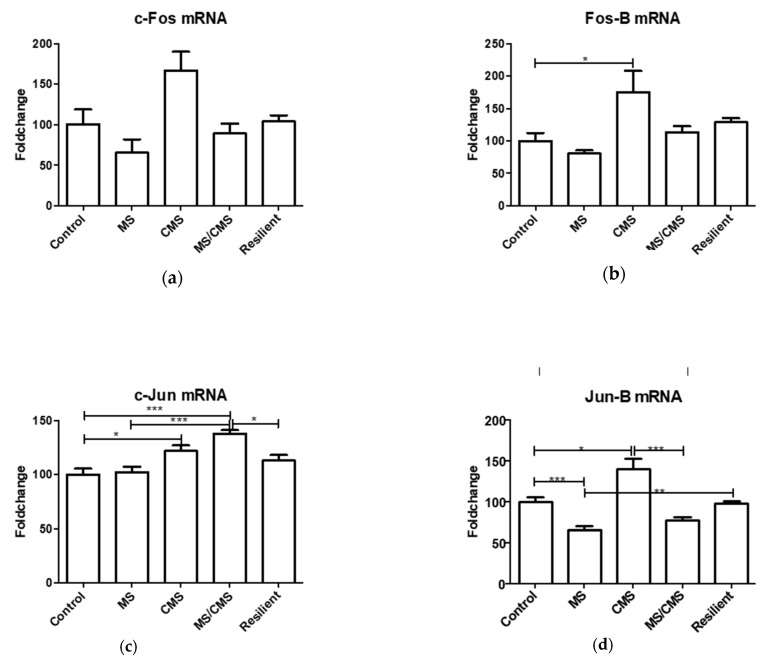
Expression of genes coding for the AP-1 complex: (**a**) c-Fos; (**b**) Fos-B; (**c**) c-Jun; (**d**) Jun-B Resilient: group of animals not exhibiting anhedonia after stress treatment. Data shown as mean fold change expression +/− SEM, normalized according to the expression of *ActB* mRNA, * = *p* < 0.05, ** = *p* < 0.02, *** = *p* ≤ 0.01.

Maternal separation and/or CMS cause either temporal or long-lasting changes in gene expression [13]. We looked at several genes relevant for neural activation and transcriptional activity such as *Creb*, *Npy*, and the activator protein-1 (AP-1) complex consisting of the immediate early genes (IEGs) *c-Fos*, *c-Jun*, *Fos-B*, *Jun-B*. The AP-1 complex can form in different ways. It can either be a homodimer of JUN family proteins or a heterodimer of JUN/FOS family proteins. It is therefore interesting that there seemed to be a concerted expression regulation across three IEGs, i.e., *c-Fos*, *Jun-B*, and *Fos-B* (Figure 2). 

**Figure 3:**
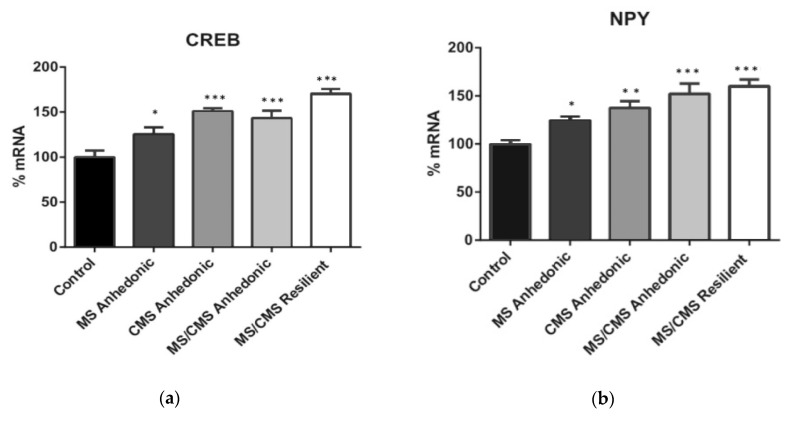
Expression of the transcription factor CREB (**a**) and of neuropeptide Y (NPY) (**b**). CREB (**a**): control vs. MS anhedonic * = *p* < 0.05; control vs. CMS anhedonic *** = *p* < 0.001; MS/CMS anhedonic vs. MS/CMS resilient * = *p* < 0.05; NPY (**b**): control vs. MS anhedonic * = *p* < 0.05; control vs. CMS anhedonic ** = *p* < 0.01. Data shown as mean fold change expression +/− SEM.

The expression levels of *Jun-B*, *Fos-B*, and *c-Fos* displayed a similar pattern in the investigated groups. They remained unchanged in both the anhedonic-like and the resilient MS/CMS groups, whereas they were decreased in the MS group and increased in the CMS group. On the other hand, the expression of *c-Jun* displayed a completely different pattern, remaining unchanged in the MS and MS/CMS-R groups and increasing in the CMS and MS/CMS-A groups. A significant 38% (*p* < 0.01) increase in *c-Jun* mRNA levels in the MS/CMS-A group (Figure 2c) might be an anhedonic trademark, since it was observed also in the CMS group, but not in the MS and the MS/CMS-R groups, which showed unaltered expression of *c-Jun*. The differential expression of *c-Jun* with respect to the other IEGs may be traced back to normal cellular physiology of the hippocampus. In this brain region, *c-Jun* is strongly expressed in the dentate gyrus, whereas none of the remaining IEGs is expressed [20]. One may therefore speculate that the variation is due to differential effects of stress on the transcriptional machinery involved in the production of AP-1 complexes. Interestingly, the *Creb* gene, coding for the constitutive transcription factor CREB, was significantly upregulated by MS and CMS as well as by their combination (Figure 3a).

CREB is involved in the transcription of *Fos*/*Jun* gene families. It is important to bear in mind that CREB can either activate or repress gene expression depending on the phosphorylation state of Ser-133 by protein kinase A (PKA) [21], being an activator in the phosphorylated state and a repressor in the dephosphorylated form [20]. We also tested the expression of *Npy*, which is independent of *Fos*/*Jun* family and codes for neuropeptide Y (NPY) involved in various physiological and homeostatic processes in the central and peripheral nervous systems. NPY antagonizes the effects of stress through multiple actions in the brain [22]. It is therefore noteworthy that *Npy* was significantly upregulated in our models, in a pattern similar to that of CREB (Figure 3b). CREB has been shown to regulate *Npy* gene expression in the amygdala [23] and hippocampus [24]. Both genes increased significantly in the anhedonic animals, but their expression did not fall to control levels in the resilient animals, suggesting that *Creb* and *Npy* expression could be involved in stress protection.

**Figure 4:**
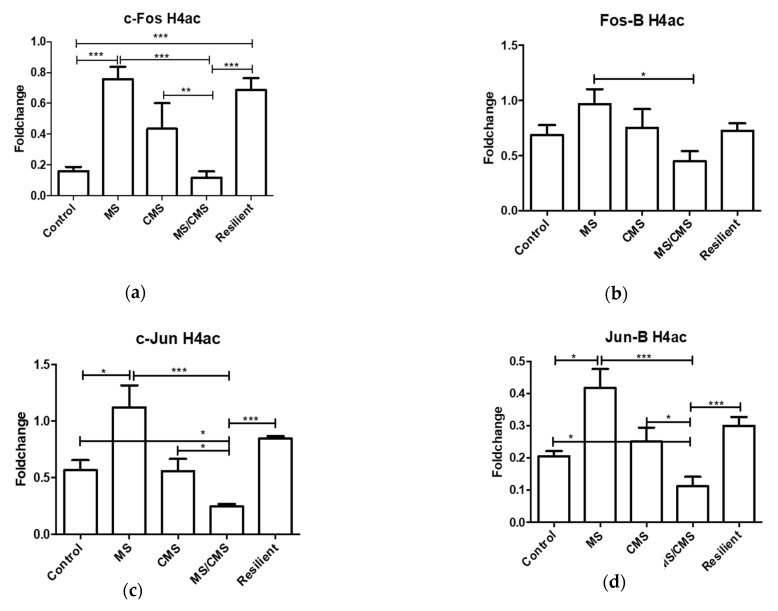
Association of histone H4 acetylation (H4ac) with different immediate early genes (IEG) promoters: (**a**) c-Fos; (**b**) Fos-B; (**c**) c-Jun; (**d**) Jun-B Results presented as fold change with respect to a control gene promoter (*ActB*). * = *p* < 0.05, ** = *p* < 0.01, *** = *p* < 0.001. Data shown as mean fold change +/− SEM.

Since MS and stress are very strong environmental factors influencing gene expression, we investigated several histone modifications to identify changes that could explain the synchronized regulation of *Fos* and *Jun*: histone H4 acetylation (H4ac), histone H3 lysine 9 trimethylation (H3K9me3), and histone H3 lysine 4 trimethylation (H3K4me3). H4ac and H3K4me3 are found on activated gene promoters, whereas histone H3K9me3 is involved in gene silencing. Two of the histone modifications, i.e., H4ac and H3K9me3, revealed specific patterns. Association between gene promoters and the activating H4ac modification was increased in the MS group for all investigated IEGs, while it was decreased in the MS/CMS-A group. Interestingly, it remained unchanged in the CMS and MS/CMS-R groups (Figure 4). 

**Figure 5:**
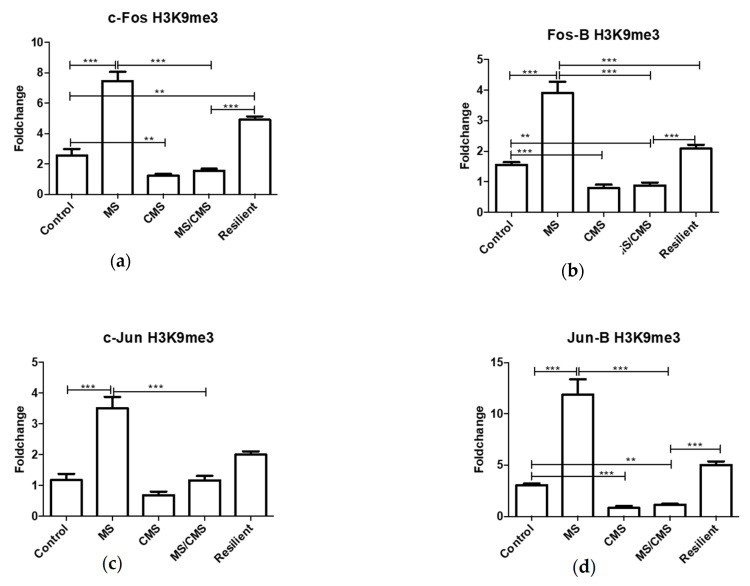
Association of histone H3 lysine 9 trimethylation (H3K9me3) with different IEG promoters: (**a**) c-Fos; (**b**) Fos-B; (**c**) c-Jun; (**d**) Jun-B Results presented as fold change over a control gene promoter (*Hist1H2BA*). ** = *p* < 0.01, *** = *p* ≤ 0.001. Data shown as mean fold change +/− SEM.

The gene expression pattern was not consistent with the H4Ac modification of the promoters, possibly due to the presence of the repressing H3K9me3 histone modification that showed a similar, yet more pronounced, pattern for all investigated *Fos*/*Jun* genes (Figure 5). The last investigated modification, H3K4me3, did not show any specific patterns that could explain the similar expression of the gene family (Supplementary Figure S1). It is clear that MS caused a massive increase in *Fos*/*Jun* expression (except for *c-Jun*) that cannot be explained by the static picture of histone modifications affecting the gene promoters. Nevertheless, a pattern could be observed suggesting that CMS had an opposite effect on the epigenetic marks compared to MS (compare Figure 4 and Figure 5). Furthermore, the levels of histone modifications related to the *c-Fos* promoter remained high in the resilient group of animals, despite *c-Fos* expression falling to the level of the control group (Figure 4a and Figure 5a) 

Numerous significant differences were observed between the anhedonic and the resilient MS/CMS groups. These may reveal mechanisms important for susceptibility to CMS, which can explain the two distinct phenotypes. Acetylation of H4 (H4ac) was consistently decreased for all IEGs except for *Fos-B* in the MS/CMS-A group. In contrast, the MS and MS/CMS-R groups showed increased association of H4ac with gene promoters (Figure 4). This could spark the idea that H4ac is an important coping mechanism for the resilient phenotype. Similar to H4Ac, the MS/CMS-R group showed increased levels of H3K9me3 than the MS/CMS-A group, except for *c-Jun* for which the change in H3K9me3 did not reach statistical significance. Interestingly, and in contrst to H4Ac, the CMS group displayed decreased association with H3K9me3, compared to controls. This decreased association may be involved in a mechanism by which CMS causes anhedonia, since both the non-anhedonic groups (MS and MS/CMS-R) displayed significantly increased association with H3K9me3. This epigenetic makeup has a clear influence on the expression of AP-1 complex genes, which were simultaneously elevated in the CMS group (Figure 2). Taken together, elevated mRNA expression of *Fos*/*Jun* family genes could be important in the establishment of the anhedonic phenotype, whereas elevated H4ac and H3K9me3 may be important for the establishment of the resilient phenotype. 

An open question in depression is whether it is a disease of the brain or a systemic disease. As our group has shown in the past [25], there is a pronounced metabolic imbalance in depressed animals. This metabolic imbalance is coupled not only to depression but also to inflammation, as shown in the same article. In this study, we can now couple epigenetic regulation not only to depression and MS-induced sensitivity to depression but also to inflammation, since the *Fos*/*Jun* gene family is not brain-specific but is expressed in many tissues. It would be interesting to investigate whether changes in gene expression can be detected in other organs, e.g., the immune system. This would be of great importance [26], as it is known that immunity is compromised in individuals with stress and depression. Furthermore, interestingly, *c-Jun* is also involved in metabolism regulation [27]. This may suggest alternative treatment options. Whether *Fos*/*Jun* expression changes in the immune system under stress and depression and whether potential expression differences could serve as a marker of predisposition to depression in the context of personalized medicine remain to be shown.

## 4. Conclusions

Exposure of rats to MS alone was enough to induce depressive-like behavior (anhedonia), measured by sucrose intake, but the application of CMS significantly increased the anhedonic behavior. A number of genes were regulated in anhedonic and resilient animals, including immediate early genes of the AP-1 complex, i.e., *c-Fos*, *c-Jun*, *FosB*, and *JunB*. Changes in AP-1 complex genes expression were associated with concerted epigenetic regulation, providing one more link between environment and gene expression for the appearance of pathology. This opens new questions for research in the field that should involve the immune system and metabolism.

## Figures and Tables

**Table 1:** Experimental groups and conditions. MS, maternal separation, CMS, chronic mild stress, MS/CMS-A: animals exposed to both CMS and MS, exhibiting anhedonia MS/CMS-R, resilient animals exposed to both CMS and MS.

	Control	MS	CMS	MS/CMS-A	MS/CMS-R
Paradigm exposure	None	MS in early life (180 min/day p2–p14)	CMS in adulthood (7 weeks of 7 mild stressors for 10–14 h/stressor)	MS in early life + CMS in adulthood	MS in early life + CMS in adulthood
Phenotype	Normal	Normal	Anhedonic	Anhedonic	Resilient
Molecular investigations N	6	6	6 (mRNA = 5)	6	6

## Supplementary Materials

The following are available online at https://www.mdpi.com/2075-4426/11/3/209/s1, Figure S1: Association of histone H3 Lysine 4 trimethylation acetylation (H3K4me3) with different IEG promoters. MS- maternal separation, CMS- chronic mild stress, MS/CMS- chronic mild stress on the MS rats, Resilient- group of animals not exhibiting anhedonia after stress treatment. Results presented as fold change over a control gene promoter +/− SEM (*ActB*). * = *p* < 0.05, ** = *p* < 0.01, *** = *p* ≤ 0.001.
