# Supplementary material for: Maternal Separation Followed by Chronic Mild Stress in Adulthood Is Associated with Concerted Epigenetic Regulation of AP-1 Complex Genes
Source: J Pers Med. 2021 Mar 16;11(3):209. doi: 10.3390/jpm11030209 (PMC8002051; doi:10.3390/jpm11030209)
Supplement: Supplementary file 1 [file jpm-11-00209-s001.pdf]

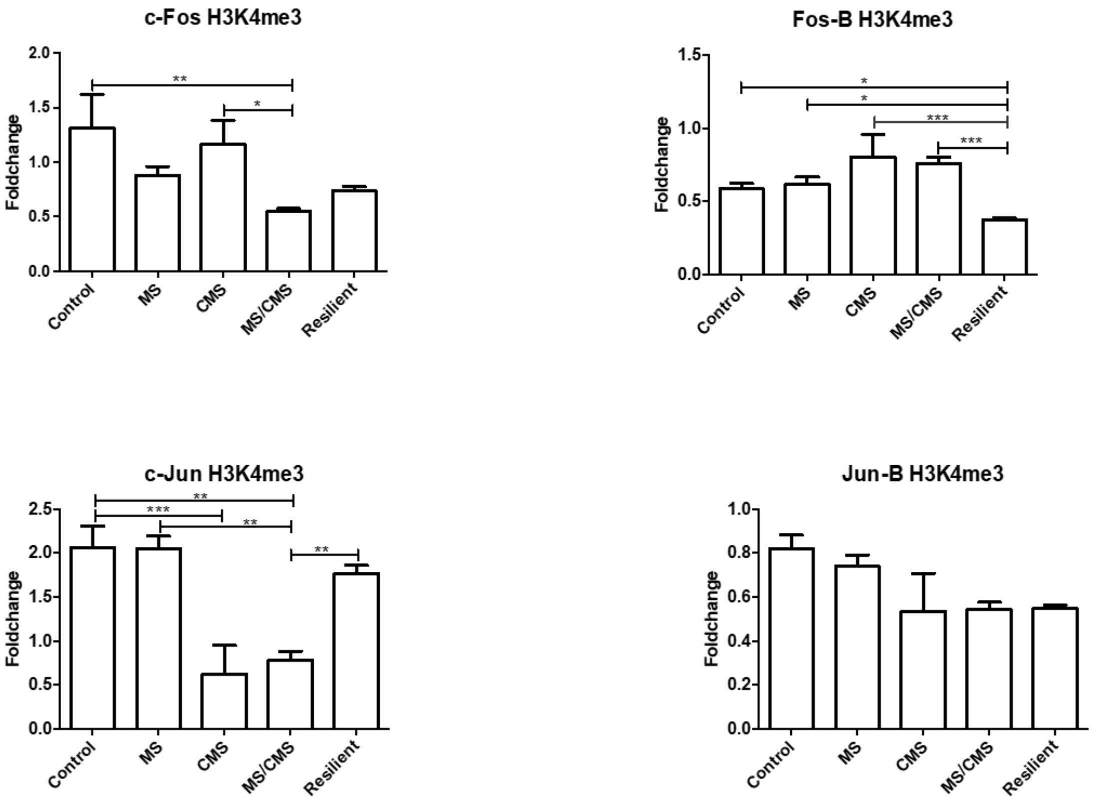

Association of histone H3 Lysine 4 trimethylation acetylation (H3K4me3) with different IEG promoters. MS- maternal separation, CMS- chronic mild stress, MS/CMS- chronic mild stress on the MS rats, Resilient- group of animals not exhibiting anhedonia after stress treatment. Results presented as fold change over control gene promoter (ActB). * = $p < 0.05$, ** = $p < 0.01$, *** = $p < =0.001$
